# Too much but less effective: Managing the cognitive load while designing the distance learning instructional formats

**DOI:** 10.30476/jamp.2020.85990.1208

**Published:** 2020-04

**Authors:** DINESH KUMAR. V, RAJASEKHAR S.S.S.N

**Affiliations:** 1 Department of Anatomy, Jawaharlal Institute of Postgraduate Medical Education and Research, Puducherry – 605006, India

**Dear Editor**

we are witnessing a paradigm shift in educational technologies due to the disruptions in academics as a result of COVID-19 outbreak. Educators across the world have begun to use technology-based applications or “just- in-time” instructional strategies to an extent never seen before. Such instructional strategies are intended to implement the curriculum in the absence of a physical classroom. However, they do not emphasize the design of instructional material used in it or the mastery of information by learner per se ( 1 ). The ultimate goal of medical education is to cause changes in behaviour through gaining information. It is vital to understand how the student’s memory handles the information load imposed by online instruction format and extent of information retention.

Information gathered through virtual learning modules is incorporated in the working memory of the learner and subsequently stored in a stipulated format ( 2 ). When a teacher delivers the lecture via streaming applications such as Zoom Cloud Meeting® or Google classroom, it is impossible to ascertain the level of attention by the student. The allotment of working memory may not be limited to grasping the information conveyed by the teacher, but also to other events happening concurrently in the surrounding environment. The information from a streaming lecture can be channelled into three sub-systems: a) visuo-spatial path, for visual images which are either projected or drawn on the screen, b) phonological path, for listening to the verbal information (delivered by the presenter), and c) a buffer system, to integrate both sub-paths into working memory ( 3 ). When the attention of the students gets bifurcated, the integration of sub-paths does not happen. Further, the frequency with which chunks of new information are presented also determines the efficacy of information consolidation. Rapid delivery of new information leads to clogging of working memory.

Secondly, the interaction between various instructional elements determines the amount of intrinsic cognitive load on working memory ( 4 ). There is a need to differentiate the instructional elements which have low interactivity compared to those with higher interactivity. For example, a lecture on anatomical components of basal nuclei has few highly interactive elements. Missing one element will not compromise on the comprehension of the other elements. However, a lecture on physiological communications and functional aspects of basal nuclei has many high interactivity elements that cannot be omitted. If the teacher omits certain interacting elements in order to make the lecture appear easy, then, it may compromise the understanding and impair learning by students. On the contrary, teaching too many essential elements at a stretch would overwhelm the cognitive capacity of the students. One method to overcome this conundrum is to create an effective cognitive schema, where multiple elements are seamlessly integrated. Also, allotting adequate time gap between different elements will enable mental rehearsal. Another method to deal with too many interacting elements in a lecture is to divide the lecture topic into 2-3 smaller segments and test the learning activity by asking the students to draw concept maps or solve multiple-choice questions. Thus, we need to understand that the primary challenge in creating content for online lectures is to manage the intrinsic load of the instructional formats.

Thirdly, in distance learning through virtual platforms, multiple factors tend to influence the cognitive load on the working memory of students. During the face to face interactions, the educator often uses attention gaining material, or purposeful distractions that break the monotony among the audience.

Such extraneous intrusions might impede the development of cognitive schemata during virtual instruction and serve as negative strategies ( 5 ). We tend to highlight three effects which would significantly influence the extraneous load in technology-based learning: a) Split-attention effect – usage of a single integrated source for presentation is better than using multiple distracting sources in the same instructional lecture, b) Modality effect – optimal utilization of multimodal input channels (visual, auditory, reading) is better when compared to unimodal input (auditory only), and c) Redundancy effect – using multiple sources for the same information could be reduced to mitigate the extraneous load ( 6 , 7 ).

Finally, the physical environment *per se* has a role in determining the processing of cognitive load in the working memory ( 8 ). Even if we consider the content, delivery and material as constant factors, the learning taking place in the conventional classroom-based physical environment would not be similar to a distance and individual learning environment. The classroom environment, with the presence of teacher and peers, is found to have a positive effect on the learner’s willingness to participate in active discussions and motivation ( 9 ). *Dowaliby and Schumer* ( 10 ) measured the level of the students’ anxiety in learner-centred and teacher-centred environments. They found that students with higher levels of anxiety performed better in the conventional teacher- centred environment compared to those with lower anxiety who performed better in a learner- centred environment. If we extrapolate this finding to virtual and distance learning, we could see that students with lower levels of anxiety could learn better.

To conclude, with the change in educational paradigms and increase in distance learning, educators need to be aware of working memory and the summative cognitive load imposed in it. The summative load, which is a combination of intrinsic and extrinsic components, can be made tolerable if scaffolding of the instructional designs is done. By sensing the processing ability of the learner, by reducing the extraneous load and tailoring the element interactivity, the inputs could be integrated into an effective mental schema and then into long term memory. Otherwise, the virtual and distance learning programs would remain as mere tools for executing the curriculum with indifference.
